# Biostimulants and their role in increasing cereal crops’ resistance to drought stress

**DOI:** 10.3389/fpls.2026.1837848

**Published:** 2026-06-01

**Authors:** Virgilija Gavelienė, Sigita Jurkonienė

**Affiliations:** Laboratory of Plant Physiology, State Scientific Research Institute Nature Research Centre, Vilnius, Lithuania

**Keywords:** antioxidant defense system, mycorrhizal fungi, plant growth-promoting rhizobacteria (PGPR), seaweed extracts, sustainable agriculture, vermicompost

## Abstract

Sustainable strategies to enhance crop resilience are increasingly needed as climate change intensifies the frequency and severity of drought events. In recent years, plant biostimulants have gained considerable attention as environmentally friendly tools for improving crop productivity and quality under water-limited conditions. Although numerous studies have reported positive effects of biostimulants, comprehensive assessments of their overall impact on cereals performance remain limited. This review presents the concept and main categories of plant biostimulants and summarizes current knowledge on their effects on plants and soil, with particular emphasis on their role in enhancing drought tolerance in major cereal crops such as wheat, barley, and other cereals. Both non-microbial biostimulants, including humic and fulvic substances, seaweed extracts, vermicompost, and plant growth regulators, and microbial biostimulants, such as PGPR and mycorrhizal fungi, are discussed with regard to their mechanisms of action. Biostimulants contribute to improved drought tolerance by enhancing antioxidant defense systems, photosynthetic efficiency, nutrient and water uptake, and phytohormonal regulation. Overall, the available evidence highlights the potential of biostimulants as sustainable tools for improving cereal productivity, grain quality, and agroecosystem resilience under drought conditions. The reviewed studies indicate that the effectiveness of biostimulants may vary among cereal species, with some crops exhibiting stronger growth and yield responses than others. In addition, this review bridges fundamental research with practical application by outlining key considerations for the effective use of biostimulants in cereal production systems.

## 1 Introduction

Drought stress is a major constraint on crop productivity and an increasing threat increasing to global food security, as it disrupts key physiological processes such as water and nutrient uptake and photosynthesis, ultimately reducing yields (Ross et al., 2020; Fahad et al., 2017). Cereals are particularly vulnerable to water deficit, and climate change induced increases in drought frequency and intensity are expected to further compromise their productivity, especially in Central and Eastern Europe (Ceglar et al., 2019; Ionita et al., 2020; Zhao et al., 2022). A bioinformatic analysis of European crop losses has shown that drought causes greater yield reductions in cereals (9% and 7.3%) than in non-cereal crops (3.8% and 3.1%) (Brás et al., 2021). Drought stress affects plants at molecular, biochemical, physiological, and morphological levels (Seleiman et al., 2021; Oguz et al., 2022), and its impact varies depending on species, developmental stage, and stress severity (Marchin et al., 2020). Drought tolerance in cereals such as wheat, barley, maize, sorghum, and rice has been widely studied (Geng et al., 2022; Batir Rusu et al., 2024). Key indicators of drought tolerance include relative water content, water-use efficiency, stomatal conductance, and photosynthetic performance (Zulfiqar et al., 2022; Guizani et al., 2024).Various strategies have been proposed to mitigate drought stress in crops; however, plant tolerance relies on complex and tightly regulated physiological and biochemical mechanisms (Hussain et al., 2018; Cao et al., 2023; Şimşek et al., 2024). Substances of natural origin that provide beneficial effects on plant growth, development, stress resistance, crop yield, and quality are referred to as biostimulants. Their physiological effects depend on their composition, which includes various organic and mineral compounds that plants can utilize as metabolites, growth regulators, and nutrients. Biostimulants comprise a diverse group of compounds, including humic substances, protein hydrolysates, amino acids, seaweed extracts, chitosan, other biopolymers, and inorganic molecules (Hassan et al., 2020; Altaf et al., 2023; Feng et al., 2024). Subgroup known as microbial biostimulants consists of beneficial microorganisms such as fungi, yeasts, and bacteria, that enhance plant growth, promote nutrient uptake, improve tolerance to abiotic stresses, and increase crop quality (European Biostimulants Industry Council, 2020). Drought t tolerance involve complex mechanisms, in which microbial metabolites such as organic acids, amino acids, proline, glycine betaine, polyamines, heat shock proteins (HSPs), dehydrins, and phytohormones play a crucial role in enhancing plant resistance to drought (Romanenko et al., 2024). Among them, proline is one of the most extensively studied metabolites associated with plant responses to abiotic stress (Hosseinifard et al., 2022; Swain et al., 2023). Additionally, γ-aminobutyric acid (GABA) has been linked to stress response regulation and improved tolerance to drought and other abiotic stresses (Ashraf et al., 2024; Palabıyık et al., 2024). Biostimulants are therefore considered promising tools for improving crop productivity in a more sustainable way, partly addressing the limitations of chemical fertilizers (Franzoni et al., 2021; Dubey et al., 2021). Despite their potential, the effectiveness of biostimulants remains inconsistent under field conditions due to high product variability and a limited understanding of their modes of action and optimal application strategies. Most studies focus on individual products under controlled conditions, whereas comparative evaluations across growth stages and environmental conditions remain limited, particularly in cereals (du Jardin, 2015; Hijri, 2023). This review therefore aims to evaluate plant and microbe-based biostimulants as tools to enhance drought tolerance in cereals, with emphasis on their physiological and biochemical mechanisms, variability of effects, and applicability under field. In addition the review discusses the composition, agronomic relevance, and modes of action of selected natural biostimulants, with particular focus on their role in improving cereal growth, yield, and drought resilience under water-limited conditions.

## 2 Drought stress and cereals

Drought stress significantly impairs crop growth, productivity, and quality, posing a major challenge to global food security. According to the European Drought Observatory (EDO), a drought warning was in effect for nearly half of the EU territory in 2022, with a red alert for 15%. By mid-January 2025, the Combined Drought Indicator (CDI) indicated warning-level drought conditions in southern Italy, the eastern Baltic Sea region, eastern Poland, central-eastern Ukraine, Greece, Cyprus, Malta, and other Mediterranean islands, as well as Ireland, northern UK, and more than a half of Turkey (NOAA National Centers for Environmental Information; 2025). Drought adversely affects the morphology and physiology of cereal crops. It impairs normal plant growth, reduces leaf expansion, stem elongation, and root development, and limits biomass accumulation across different growth stages. In addition, it disrupts both water relations and reduces water-use efficiency (Kızılgeçi et al., 2017; Naeem et al., 2024; Ali et al., 2025). At the physiological level, drought alters hormonal balance, typically increasing abscisic acid levels while reducing cytokinins, which induces stomatal closure and restricts gas exchange (Wach and Skowron, 2022). Reduced stomatal conductance limits CO_2_ uptake, thereby decreasing photosynthetic activity and ultimately reducing yield (Wahab et al., 2022). In addition, drought stress promotes the accumulation of reactive oxygen species (ROS), leading to oxidative damage to cellular components, including membranes, proteins, and nucleic acids (Shayen et al., 2023a). This cascade of morphological and physiological changes ultimately reduces crop productivity. For instance, drought has been estimated to reduce wheat yields in European countries by up to 10% (Webber et al., 2018). Yield losses are primarily associated with reductions in grain number, grain size, and overall biomass production. The magnitude of these effects depends on crop species, genotype, developmental stage, and the severity and duration of drought stress.Cereal crops exhibit considerable variation in drought tolerance. Although barley is generally more tolerant than wheat, it remains vulnerable under prolonged drought and elevated temperature conditions (Szczepanek et al., 2018). Genotypic variability plays a key role, as some cultivars show better adaptation to water-limited environments (Mega et al., 2023). Additionally, the developmental stage during which drought occurs is critical. Plants are particularly susceptible during reproductive stages, when prolonged water deficits can negatively impact grain setting, flower production, seed composition, and seed viability (Dufková et al., 2020). Drought can also negatively affect crop quality. For example, water deficits have been shown to reduce oil content in rapeseed. Understanding the impact of drought on cereal production and more importantly, developing sustainable and effective strategies remains a key challenge for agricultural scientists (Camaille et al., 2021; Begna, 2024). To mitigate these effects, several approaches have been explored to enhance drought resistance in cereals, including plant breeding, genetic engineering, and biotechnology-based tools (Nuccio et al., 2018; Villegas-Escobar, 2024). However, these methods can be time-consuming, costly, or potentially harmful to the environment, thus prompting the search for alternative strategies. Recently, biostimulants have gained attention as promising tools to enhance cereal tolerance to abiotic stress. Compounds such as plant growth regulators, amino acids, probiotics, and other biostimulants have been applied to improve cereals plant recovery and growth under drought conditions; Lalarukh et al., 2022).

## 3 Biostimulant categories

**Figure 1:**
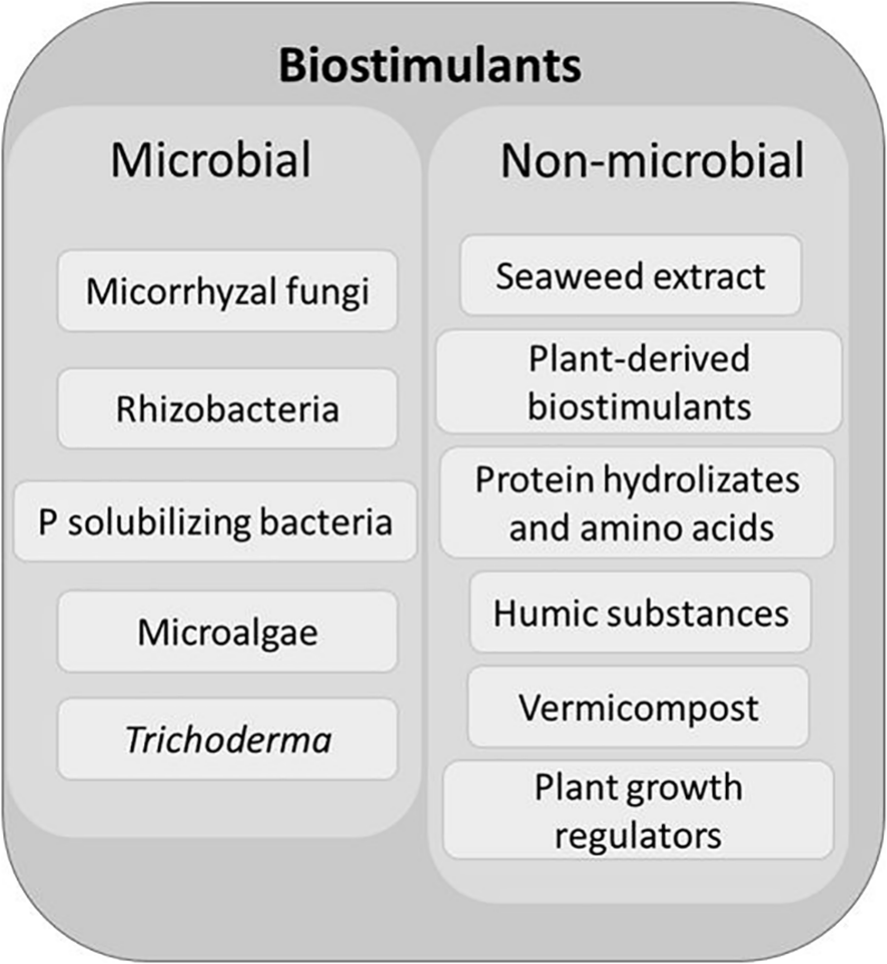
Classification of biostimulants.

Biostimulants are substances/micro-organisms that can stimulate plant growth, nutrition and stress tolerance independently of their nutritional content. They are increasingly considered as alternatives to chemical fertilizers, which are associated with adverse environmental impacts (Boutahiri et al., 2024). Also referred to as bioeffectors, bioprotectors, or bio-based products, biostimulants comprise a wide range of organic and inorganic compounds as well as beneficial microorganisms that promote plant productivity and stress resilience (du Jardin, 2015; Franzoni et al., 2022; Mandal et al., 2023; Jiang et al., 2024). They can be applied to crops through foliar fertilization, fertigation, or direct soil application, improving plant growth and performance under both optimal and stress conditions, quality (Van Oosten et al., 2017). Plant biostimulants contribute to sustainable agriculture by promoting plant growth and mitigating the detrimental effects of sub-optimal environmental conditions. Unlike chemical fertilizers, biostimulants do not exert negative impacts on ecosystems (Boutahiri et al., 2024; Garcia-Caparros et al., 2025). Their use is typically associated with improved nutrient use efficiency, increased tolerance to abiotic stress, and enhanced crop quality. Biostimulants are commonly classified into two main categories: (1) non-microbial biostimulants, (e.g., seaweed extracts, vermicompost, humic and fulvic acids, plant growth regulators, protein hydrolysates, amino acids, and (2) microbial biostimulants, such as arbuscular mycorrhizal fungi (AMF), PGPR (Rouphael et al., 2020; du Jardin et al., 2020) (**Figure 1**). Both groups can modulate primary and secondary plant metabolism, leading to enhanced antioxidant activity and improved stress adaptation (Papa et al., 2022; Ciriello et al., 2025). However, the strength of evidence supporting their effectiveness under drought stress varies substantially among biostimulant classes. Current literature indicates that non-microbial biostimulants particularly seaweed extracts and humic substances the most consistent and reproducible effects on crop yield and quality under drought conditions. These compounds primarily regulate physiological processes such as hormonal balance, osmotic adjustment, antioxidant activity, and membrane stability, which are directly linked to improved drought tolerance (Gupta et al., 2022; Rouphael et al., 2020). Their widespread commercial availability and relatively predictable performance across different environments further support their practical applicability (Arioli et al., 2024; Iqbal et al., 2022). In contrast, microbial biostimulants such as AMF and PGPR demonstrate strong potential for enhancing drought resilience through improved nutrient acquisition, root system architecture, and soil–plant interactions (Ganugi et al., 2021; Pal et al., 2024). Nevertheless, their effectiveness is often more variable and context-dependent, as it is influenced by soil conditions, microbial survival, and plant genotype. As a result, while microbial inoculants can significantly improve crop productivity and quality, especially under stress conditions, the consistency of their field performance remains less certain compared to non-microbial products. Recent advances suggest that combined applications of microbial and non-microbial biostimulants may provide the most promising strategy for enhancing drought tolerance. Synergistic interactions between bioactive molecules and beneficial microorganisms have been shown to improve plant growth, yield, and metabolic responses more effectively than individual treatments (Castiglione et al., 2021; Wadduwage et al., 2024; Benito et al., 2025). Such combinations have great potential to improve both plant productivity and nutritional status, as well as to modulate plant metabolomes for the development of novel crop functionalities. For example, the combination of humic biostimulants with microbial inoculants has been reported to enhance lettuce productivity, nutrient uptake, and primary and secondary metabolism (Savarese et al., 2022). Although the benefits of biostimulants are well documented, knowledge about their effects especially those of probiotic-based products on cereal growth, productivity, and yield quality remains limited. Therefore, further investigation is required to assess the influence of biostimulants on cereals and to integrate them into environmentally sustainable agricultural practices. Identifying biostimulants that promote growth and yield in crops such as wheat, oats, barley, and rice, while minimizing environmental impact, represents a promising direction for future crop production strategies. Functional characterization of biostimulant properties and mechanisms of action is crucial for understanding the complex interactions among biological entities within diverse agroecosystems. Even incremental advances in this field may result in significant benefits for industrial applications, ecological intensification, and the sustainability of global food and bioresource systems. Thus, by reducing the adverse effects of excessive chemical inputs, biostimulants represent a sustainable strategy for mitigating environmental threats to crop productivity. Their use improves soil fertility, promotes crop production, and supports the development of sustainable agricultural systems.

## 4 Seaweed extracts

One of the most promising classes of biostimulants is seaweed extracts (SE), which contain a wide range of growth-promoting substances such as cytokinins, auxins, gibberellins, and other macro- and micronutrients essential for plant growth and development (Mughunth, 2024; El Bzar et al., 2026). Due to their compatibility with both conventional and organic farming systems, SEs are widely used as biostimulants; however, their effects on crop performance are not uniform and depend on multiple interacting factors (Ali Omar et al., 2021). Seaweeds are categorized into three main groups based on their pigmentation: Phaeophyta (brown), Rhodophyta (red), and Chlorophyta (green). Among these, the most used seaweeds in agriculture are brown seaweeds, including species of the genera *Ascophyllum*, *Fucus*, and *Laminaria* (Sariñana-Aldaco et al., 2025). Seaweed extracts improve plant growth at all stages, including germination, harvest, and post-harvest. Their effectiveness depends on factors such as the type of seaweed, plant species, concentration, and extraction method (Quitério et al., 2022). Seaweed-derived biostimulants hold great promise due to their ability to: (a) be effective at very low concentrations, (b) enhance crop resistance to abiotic stress, and (c) boost protein production in protein-rich plants (Alaila and Elsalhin, 2022). Also, Kocira et al. (2020b) studies report that seaweed biostimulants can be more effective (early seed germination, root growth, and overall crop productivity) when combined with other bioactive compounds. Several studies indicate that SE application can reduce relative water loss, alleviate leaf wilting, and support recovery from short-term drought stress (4–10 days) in wheat (Shukla et al., 2018, 2019; Sharma et al., 2019). These physiological responses are often associated with enhanced antioxidant activity, which helps mitigate oxidative damage caused by reactive oxygen species under stress conditions (El Boukhari et al., 2020). However, other studies report no significant improvements in yield or even negative effects on germination under certain stress scenarios, highlighting the importance of context-dependent responses. Under severe drought conditions, SE-treated plants may perform better than untreated controls, but overall productivity typically remains constrained by stress intensity (Murtic et al., 2018; Trivedi et al., 2018; Hashem et al., 2019). This suggests that SEs should be viewed primarily as stress-mitigating agents rather than yield-enhancing inputs under all conditions. An additional source of variability arises from the raw material itself. The biochemical composition of seaweeds fluctuates depending on environmental conditions, growth stage, and genotype, with documented seasonal variation in polysaccharides, polyamines, lipids, and proteins (Fletcher et al., 2017). Commercial products may also contain mixed seaweed species due to non-selective harvesting practices, further complicating standardization and reproducibility (Deolu-Ajayi et al., 2022). SEs can be applied via foliar sprays or soil treatments and have been shown to influence plant metabolism, root development, and biomass accumulation. Nevertheless, much of the available evidence is derived from controlled experiments, and data on cereal crops particularly regarding yield stability and grain quality under field conditions remain limited and sometimes contradictory (Carillo et al., 2020; Barone et al., 2018). However, there is limited data on the effects of algae on cereal growth and yield quality, and conclusions remain contradictory. In recent years, research has increasingly focused on evaluating the effects of different concentrations of seaweed algae on cereal growth and development, as well as their potential to mitigate drought stress, through the assessment of growth parameters and various physiological responses (Hamouda et al., 2022; lharbi et al., 2022; Sherif et al., 2025 For example, specific application rates (e.g., 0.5 g SE) have been reported to improve wheat performance under drought conditions; however, such findings are highly context-specific and should not be generalized without considering environmental and agronomic conditions. In some cases, enhanced effects have been observed when SEs are applied in combination with other bioactive compounds, suggesting potential (Lalarukh et al., 2022) synergistic interactions (Kocira et al., 2020a; Lalarukh et al., 2018; Shayen et al., 2023b). However, these combined approaches remain insufficiently standardized for broad agronomic recommendations.

## 5 Plant-derived biostimulants

Plant-derived biostimulants, such protein hydrolysates (PHs) and osmocompatible solutes (OCS), represent a heterogeneous group of substances capable of enhancing crop productivity and modulating plant responses to abiotic stress (Caruso et al., 2019). These compounds include phytohormones, flavonoids, amino acids, protein hydrolysates, and complex plant extracts. Their primary mode of action is associated with the regulation of physiological and molecular processes rather than the direct provision of nutrients (du Jardin, 2015; Franzoni et al., 2021; Mandal et al., 2023). Under drought conditions, one of the key plant response mechanisms involves the regulation of reactive oxygen species (ROS). Biostimulants can enhance the activity of antioxidant enzymes such as superoxide dismutase, catalase, and peroxidases, thereby reducing oxidative damage at the cellular level. This is particularly important for cereals, whose productivity is highly sensitive to water deficit during critical growth stages (Hasanuzzaman et al., 2020). However, these effects are often less consistent under field conditions due to environmental variability and genotype-specific responses (Ma et al., 2022). Protein hydrolysates consist of mixtures of peptides, oligopeptides, and amino acids obtained through chemical, thermal, or enzymatic hydrolysis of plant- or animal-derived raw materials. They are considered promising tools for improving plant tolerance to drought stress, as they can regulate metabolic processes, enhance osmotic adjustment, and strengthen antioxidant defense systems (Nardi et al., 2016; Colla et al., 2017). Positive effects on biomass accumulation and photosynthetic efficiency have been reported, particularly under stress conditions; however, the underlying mechanisms in cereal systems are not yet fully elucidated. Moreover, variability in raw materials and production processes may lead to differences in product composition, affecting their consistency and efficacy. Osmocompatible solutes (OCS) are low-molecular-weight organic compounds that accumulate in plant cells without causing cellular damage. They play a crucial role in osmoregulation by maintaining cell turgor and water potential, thereby mitigating the effects of water deficit (Colla et al., 2017). OCS can enhance water uptake, regulate stomatal conductance, and improve overall plant water status under drought conditions. In addition, they contribute to membrane stabilization and reduce ROS accumulation, providing protection against multiple abiotic stresses, including heat and cold (García-Segura and Pardos, 2018). The use of OCS is also associated with economic and environmental benefits. They may reduce the need for chemical fertilizers and pesticides by enhancing plant resilience, while improving water and nutrient use efficiency and potentially increasing yields even under non-stress conditions. As these compounds are typically derived from renewable resources, their environmental footprint is relatively low. However, their effectiveness depends on multiple factors, including stress intensity, plant species, formulation, and application strategy. Complex plant extracts represent another important category of biostimulants, often containing multiple bioactive compounds that may act synergistically to regulate plant metabolism and stress responses. Nevertheless, the identification of specific active components and their mechanisms of action remains limited, complicating the interpretation and comparison of experimental results across studies (Calvo et al., 2014).A notable limitation in current research is the relatively small number of comparative studies evaluating different classes of plant-derived biostimulants under similar experimental conditions. This limits the ability to distinguish generalizable effects from context-specific responses. Furthermore, interactions with environmental factors, soil properties, and crop genotypes are not always systematically addressed. Overall, plant-derived biostimulants, protein hydrolysates, and osmocompatible solutes show considerable potential for mitigating drought stress in cereal crops by influencing multiple physiological and biochemical pathways. However, further research is required to clarify their mechanisms of action, ensure consistent performance under field conditions, and optimize their application across different cereal species and agroecosystems.

## 6 Humic substances

Humic substances (HSs) are complex organic compounds formed through the decomposition of plant and animal residues. Depending on the extraction method, HSs can be obtained from soil, vermicompost, and low-rank coals such as lignite, brown coal, and leonardite (Fatima et al., 2021). Their biochemical activity is highly variable and largely determined by both their origin and extraction method, which complicates direct comparisons among studies and partly explains inconsistent experimental outcomes (García et al., 2019). Structurally, HSs are regarded as supramolecular associations of small, heterogeneous molecules stabilized by weak intermolecular forces, which underpins their multifunctional role in plant-soil systems. In cereal cultivation, humic acids (HAs) play a significant role in improving soil quality and plant physiological performance, particularly under drought stress conditions. HAs enhance soil physical and biochemical properties by improving aggregation, water-holding capacity, and microbial activity, thereby creating more favorable conditions for root growth and water uptake (Nardi et al., 2021). However, the magnitude of these effects depends strongly on soil type and environmental conditions. For example, improvements in water retention are more pronounced in coarse-textured soils, whereas in clay-rich soils the benefits may be less evident. This suggests that HS application should be context-specific rather than universally recommended. Additionally, HAs increase the availability of macro- and micronutrients through chelation and co-transport mechanisms, which is especially important for wheat grown in nutrient-limited or drought-affected soils (Yang et al., 2021). HSs also act as plant biostimulants by influencing endogenous hormone-like activity, particularly auxins and cytokinins, which regulate root architecture, leaf development, and stress responses in wheat. These hormonal effects contribute to improved nutrient metabolism, enhanced photosynthetic efficiency, and increased tolerance to water deficit (Canellas et al., 2020; Nardi et al., 2021; Biswas et al., 2025). Moreover, HSs modulate key metabolic processes in wheat, including respiration, protein synthesis, and enzyme activity, supporting plant growth under suboptimal moisture conditions (Ahmad et al., 2018) (**Table 1**). At the metabolic level, HSs have been shown to modulate respiration, protein synthesis, and enzyme activity, contributing to improved stress tolerance (Ahmad et al., 2022). Under drought stress, they also play a role in maintaining redox homeostasis and reducing oxidative damage, thereby sustaining photosynthetic activity (Picault et al., 2009; Trevisan et al., 2011; Ren et al., 2021). Despite these observations, the underlying molecular mechanisms are still not fully resolved, and further research is needed to link gene expression changes with physiological outcomes. HS’sare traditionally classified into humic acids, fulvic acids, and humins based on their solubility and molecular weight (De Melo et al., 2016). Among these, humic acids are most commonly used in agricultural applications due to their stability and functional group composition (Ampong et al., 2022). HAs are high-molecular-weight compounds rich in phenolic (–OH) and carboxylic (–COOH) functional groups, soluble in alkaline media and precipitating under acidic conditions (Nardi et al., 2021). In contrast, fulvic acids have lower molecular weights and are more mobile and potentially more rapidly available to plants (Schellekens et al., 2017). Comparative studies suggest that combined applications of humic and fulvic fractions may provide more consistent benefits than individual fractions alone, although this area remains underexplored. Commercial HS-based biostimulants used in wheat production are commonly extracted from leonardite, a humified plant-derived material representing an intermediate stage between peat and lignite. These products are widely applied to enhance root development, nutrient uptake, and drought resilience in wheat crops. Numerous studies have demonstrated that humic acid applications positively modulate wheat growth, photosynthesis, hormonal balance, and osmolyte accumulation, resulting in improved biomass production and grain yield under drought stress (Chen et al., 2022; Ma et al., 2022) (**Table 1**). Similarly, the application of humic and fulvic acids has been shown to improve of spring wheat nutrient status and yield across different soil types (Khan et al., 2018; Brazienė et al., 2021). In this way it can be said that humic substances are multifunctional biostimulants with applications in sustainable agriculture.

**Table 1:** Biostimulant effects on cereal crops under drought stress.

Category	Biostimulant	Crop	Main effects	Reference
Non-microbial	Seaweed extract	*Triticum aestivum*	↑ growth; ↑ tolerance	Sherif et al., 2025
Non-microbial	Humic acid	*Zea mays*; *T. aestivum*	↑ IAA; ↑ osmolytes; ↑ photosynthesis	Chen et al., 2022; Ahmad et al., 2018
Non-microbial	Vermicompost	*Zea mays*	↑ growth; ↑ antioxidants	Ahmad et al., 2022; Rehman et al., 2023
Non-microbial	GABA	*T. aestivum*; *Z. mays*	↓ oxidative damage; ↑ RWC	Shaw et al., 2016; Zhao et al., 2023
Non-microbial	IAA	*Oryza sativa*	↓ ROS; ↓ lipid peroxidation	Sharma et al., 2018
Non-microbial	GA (priming)	*Z. mays*; *T. aestivum*	↑ germination; ↑ yield	Ghobadi et al., 2012; Salih and Tuncturk, 2020; Xie et al., 2019
Non-microbial	Proline	*Z. mays*; *T. aestivum*; *O. sativa*	↑ osmotic adjustment; ↑ antioxidants	Khan et al., 2025; Farooq et al., 2017; Li et al., 2024; Hanif et al., 2022
Microbial	PGPR	*T. aestivum*; *A. sativa*; *H. vulgare*	↑ RWC; ↑ photosynthesis; ↑ yield	Gavelienė and Jurkonienė, 2022; Slimani et al., 2023
Microbial	PGPM + Ca	*T. aestivum*	↑ water balance; ↑ physiology	Zareyan et al., 2025
Microbial	*Azotobacter* spp.; *Bacillus* spp.	*T. aestivum*	↑ productivity; ↑ tolerance	Zareyan et al., 2025
Microbial + Non-microbial	PGPR + Vermicompost	*T. aestivum*	↑ RWC; ↑ productivity; ↓ ROS	Kanwal et al., 2017; Talaat and Abdel-Salam, 2024a
Microbial + Non-microbial	Humic acid + Seaweed + PGPM	*T. aestivum*	↑ growth; ↑ nutrient uptake	Pathak et al., 2024

Summary of microbial and non-microbial biostimulants improving growth, physiological traits, and stress tolerance in cereals.

Arrows indicate direction of change (↑ increase; ↓ decrease).

## 7 Vermicompost

Interest in vermicompost has increased substantially in recent years due to its well-documented role in improving plant performance under abiotic stress, particularly drought in cereal crops. Vermicompost is the end product of the biological degradation of organic matter by earthworms in synergy with microorganisms, representing a sustainable approach to recycling organic residues while enhancing soil quality (Pooja et al., 2022). Vermicompost is rich in biologically active compounds, including plant growth regulators such as auxins, cytokinins, and humic substances, which are produced through microbial activity during the decomposition process. As a result, vermicompost not only facilitates organic waste recycling but also alleviates the environmental burden associated with conventional waste management practices (Hussain et al., 2018). Its application plays a crucial role in sustainable agriculture by providing an environmentally friendly and cost-effective method for managing organic agricultural residues (Dreslova, 2023). From a soil science perspective, vermicompost significantly improves soil structure by promoting the formation of stable microaggregates (coprolites), which enhance soil porosity and water-holding capacity key factors for maintaining plant water status under deficit conditions microbial activity (Aksakal et al., 2016). In addition, vermicompost improves soil cation exchange capacity, facilitating nutrient retention and availability, which supports more efficient uptake of essential macro and micronutrients by cereal roots nutrients This improvement contributes to higher grain yield and quality. The slow-release nature of vermicompost ensures a continuous nutrient supply throughout the growing season, thereby reducing reliance on synthetic fertilizers and minimizing nutrient losses to the environment (Oyege and Bhaskar, 2023; Rehman et al., 2023). Consequently, cereals cultivated with vermicompost tend to exhibit enhanced vigor, stress resilience, and productivity (Joshi et al., 2015; Walia and Kaur, 2024). Moreover, soil application of wheat straw derived vermicompost at rates of 6–8 t ha^-^¹ significantly improved wheat growth and drought resilience by increasing root and shoot length (up to ~28%), biomass accumulation (up to ~50%), photosynthetic performance, and antioxidant enzyme activities, including superoxide dismutase (SOD), peroxidase (POD), and catalase (CAT) (Ahmad et al., 2022) (**Table 1**). These effects are primarily attributed to improved soil moisture retention, enhanced nutrient availability, and reduced oxidative damage under drought stress. Beyond soil improvement, vermicompost also exhibits pronounced biostimulatory properties, enhancing nutrient uptake efficiency and tolerance to abiotic stress factors in cereals (Rehaman et al., 2023) (**Table 1**). Its filtrates can be applied either as soil drenches or foliar sprays; however, foliar application requires appropriate dilution to prevent potential phytotoxicity caused by excessive nutrient concentrations. In organic farming systems, the guiding principle is to “feed the soil rather than the plant, “ with an emphasis on improving soil structure, biological activity, and long-term fertility. Vermicompost supports beneficial microbial communities that decompose organic matter and gradually release nutrients required for plant growth. Due to its high nutrient density, vermicompost application should not exceed 25% of the soil volume (Blouin et al., 2019; Aslam et al., 2023). The results reported by Talaat and Abdel-Salam (2024b) further demonstrate that the combined use of vermicompost and effective microorganisms significantly enhanced wheat growth and productivity while reducing drought-induced oxidative stress by limiting the accumulation of superoxide anion radicals and hydrogen peroxide Similarly, synergistic effects have been observed in maize, where vermicompost application improves photosynthetic efficiency, nitrogen metabolism, and antioxidant capacity under drought stress (Rehman et al., 2023). Despite these promising findings, comprehensive understanding of the mechanisms through which vermicompost alleviates drought stress in cereals remains limited, particularly with respect to its influence on growth regulation, physiological processes, and enzymatic antioxidant systems.In particular, further research is needed to clarify its effects on growth regulation, physiological responses, and antioxidant enzyme systems at the molecular and biochemical levels. Future studies should prioritize cereal-specific responses, optimize application rates and methods under water-limited conditions, and evaluate long-term impacts on soil functionality and crop productivity.

## 8 Plant growth regulators

Plant growth regulators (PGRs) represent a functionally diverse group of biostimulants that modulate plant responses to drought stress through hormonal regulation of growth, metabolism, and stress signaling (Ullah et al., 2018; Kosakivska et al., 2022). However, their effectiveness is highly context-dependent, varying with plant genotype, stress intensity, application timing, and environmental conditions (Ullah et al., 2018; Kamruzzaman et al., 2022). Therefore, optimizing PGR application methods is essential for enhancing their efficiency while minimizing environmental impacts and economic costs. To withstand drought stress, plants activate a wide range of morphological, physiological, and biochemical responses that support growth and maintain productivity. Considerable scientific attention has been given to seed priming and foliar application of phytohormones aimed at improving cereal tolerance to environmental stressors (Iqbal et al., 2022). Phytohormones act as key chemical messengers regulating plant growth, development, and metabolism at various growth stages. Auxins enhance root development, thereby improving water uptake under drought conditions, whereas gibberellins (e.g., GA_3_) are primarily effective during early growth stages, particularly through seed priming, which improves germination and early seedling vigor. Exogenous IAA has been shown to enhance drought resistance in cereals by increasing antioxidant activity, as well as carbohydrate and protein content, ultimately promoting biomass accumulation (Muhammad et al., 2016). For example, in rice, exogenous IAA reduced lipid peroxidation and ROS accumulation in spikelets under drought and high-temperature stress (Sharma et al., 2018) (**Table 1**). Studies demonstrate that wheat seeds primed with GA_3_ show higher germination percentage, faster germination rate, and improved growth and yield in crops such as wheat, maize, and sorghum (Ghobadi et al., 2012; Salih and Tuncturk, 2020) (**Table 1**). In maize, GA improves drought tolerance by maintaining membrane integrity and increasing chlorophyll content, relative water content, and macronutrient levels (Xie et al., 2019) (**Table 1**). Other PGRs, including salicylic acid, also contribute to improved drought tolerance through the activation of antioxidant defense systems (Pei et al., 2019). Cytokinins and ethylene exhibit more complex, sometimes contrasting roles: cytokinins delay senescence and sustain photosynthetic activity, whereas ethylene often associated with stress signaling can either enhance tolerance (e.g., via proline biosynthesis and antioxidant activation) or accelerate senescence depending on concentration and timing Mukherjee, 2020; Desta and Amare, 2021). For example, in wheat, ethylene was shown to regulate proline biosynthesis and strengthen antioxidative mechanisms during heat stress. Application of 200 µL ethephon and 50 mM proline markedly enhanced heat tolerance by activating defense mechanisms and protecting photosynthetic pigments via upregulation of photosynthesis-related genes (Sehar et al., 2023). Similarly, growth retardants such as paclobutrazol shift resource allocation from vegetative growth toward reproductive development, which may improve yield stability under drought but can limit biomass production under non-stress conditions (Mukherjee, 2020; Desta and Amare, 2021). Overall, the comparative analysis indicates that PGRs do not act through isolated pathways but rather through coordinated regulation of water relations, redox homeostasis, and carbon metabolism. Their practical effectiveness therefore depends on aligning the specific physiological role of each regulator with the timing and intensity of drought stress. In recent years, considerable attention has been directed toward the use of osmoregulatory compounds (osmoprotectants) to enhance drought resistance in cereals. In contrast to PGRs, which primarily act through signaling pathways, osmoprotectants contribute more directly to cellular stabilization under drought stress. These compounds such as proline and γ-aminobutyric acid (GABA) share key physicochemical properties (small size, high solubility, low toxicity) that enable them to maintain cellular homeostasis without disrupting metabolism (Hossain et al., 2019). Their advantages include high efficiency, simplicity of use, low cost, and the absence of a need for specialized equipment. Proline is among the most widely studied and effective osmoprotectants, naturally accumulating in plants under drought stress (Siddique et al., 2018). A synthesis of available studies shows that proline simultaneously stabilizes membranes, protects macromolecules, enhances antioxidant capacity, and supports photosynthetic efficiency. Importantly, its function appears to be both protective during stress and restorative during recovery phases (Kaur and Asthir, 2017; Hossain et al., 2019; Hosseinifard et al., 2022; Jurkonienė et al., 2023). Exogenous proline increases the concentration of osmolytes and antioxidants, promoting stress tolerance by maintaining osmotic balance, reducing reactive oxygen species (ROS), and stabilizing cellular macromolecules (Siddique et al., 2018). Its application in wheat enhances osmotic protection under water deficit and promotes the accumulation of high levels of chlorophyll, proline, glycine betaine, and soluble phenols (Farooq et al., 2017; Hosseinifard et al., 2022) (**Table 1**). The main application methods include seed soaking and foliar spraying. In maize (*Zea mays*), foliar proline application (30 mM) enhances drought resistance by improving growth parameters, increasing chlorophyll content, reducing electrolyte leakage and oxidative stress indicators (H_2_O_2_, MDA), and elevating protein and macronutrient (N, P, K) levels. Such responses facilitate improved metabolic activity and osmotic regulation (Khan et al., 2025) (**Table 1**). Smilarly alleviate the adverse impacts of osmotic stress on rice-seeding growth (*Oryza sativa*) (Hanif et al., 2022) (**Table 1**). In wheat, proline application regulates osmolyte content, chlorophyll levels, antioxidant enzyme activity, water-holding capacity, and tissue structure of flag leaves, ultimately mitigating yield losses under drought stress (Li et al., 2024) (**Table 1**). Although the mechanistic relationship between proline accumulation and stress tolerance remains under investigation, numerous studies confirm that proline accumulation is beneficial, particularly during post-stress recovery (Kaur and Asthir, 2017; Hosseinifard et al., 2022: Jurkonienė et al., 2023). However, responses to proline are not uniform. Its effectiveness is strongly influenced by stress intensity and species-specific metabolism, indicating that proline accumulation is not merely a stress marker but part of a dynamic regulatory network. This nuance often becomes evident only in cross-study comparisons (Schepers et al., 2024; Khamis et al., 2025). The non-protein amino acid γ-aminobutyric acid (GABA) is increasingly recognized as a key regulator of plant responses to abiotic stresses, including drought. While proline primarily contributes to osmotic balance, GABA functions at the interface of metabolism and signaling. It modulates carbon and nitrogen metabolism, supports mitochondrial function, and regulates antioxidant enzyme systems (Vijayakumari et al., 2016; Hayat et al., 2023). For example in wheat, GABA reduced drought-induced oxidative damage and increased phenolic compound content, demonstrating enhanced radical scavenging activity, with 1.0 mM being the most effective concentration (Zhao et al., 2023) (**Table 1**). It is also reported that GABA induced drought tolerance in maize through the jasmonic acid pathway by activating antioxidant defense mechanisms and abscisic acid synthesis (Shaw et al., 2016) (**Table 1**). Also, chitosan stimulates drought tolerance in crops like maize (Rabêlo et al., 2019) and barley (Hafez et al., 2020). This treatment augmented antioxidant mechanisms, photosynthesis, and grain yield. A key integrative insight is that proline and GABA share a common precursor (glutamate), which partially explains their synergistic effects when applied together (Brikis et al., 2018). Experimental evidence supports this interaction, showing enhanced survival and stress tolerance when both compounds are used in combination rather than individually (Jurkonienė et al., 2025). This suggests that future strategies should move beyond single-compound applications toward combined treatments targeting multiple stress-response pathways. Taken together, the evidence indicates a fundamental distinction between PGRs and osmoprotectants: PGRs primarily regulate stress responses at the signaling and developmental level, whereas osmoprotectants act at the cellular and biochemical level to stabilize plant function. Despite these differences, both groups converge on key outcomes of photosynthesis, reduction of oxidative damage, and preservation of water status.From a practical standpoint, the most effective drought mitigation strategies are likely to involve their combined use. PGRs can optimize developmental responses and stress signaling, while osmoprotectants provide immediate cellular protection. Future research should therefore prioritize integrated approaches, dose optimization, and crop-specific protocols rather than isolated compound testing.

## 9 Microbial biostimulants

Microbial biostimulants represent the largest and and fastest-growing categories of biostimulant products currently used in agriculture. These preparations contain beneficial microorganisms that may contribute to drought stress mitigation through a range of mechanisms, including osmotic adjustment, improvement of root system architecture, modulation of phytohormone balance, activation of antioxidant defense systems, and regulation of stress-responsive gene expression (Caradonia et al., 2019; Mikiciuk et al., 2024). However, the magnitude and consistency of these effects depend strongly on crop species, environmental conditions, soil characteristics, and application strategies. Ecological and agricultural innovations based on microbial biostimulants have demonstrated considerable potential to enhance drought resilience in crop production systems. Microbial plant biostimulants may consist of single microbial strains or consortia, commonly including species of *Azotobacter*, *Rhizobium*, *Azospirillum*, PGPR, and AMF (Alori and Babalola, 2018; Shirinbayan et al., 2019). These products are generally based on viable microorganisms (probiotics) and are typically produced using fermentation and formulation. When applied to seeds, soil, or plant surfaces, they can influence plant growth, through improved nutrient cycling, enhanced nutrient availability, and modulation of plant microbe interactions (Kumar et al., 2022). Nevertheless, reported outcomes vary widely depending on microbial strain compatibility with the host plant and environmental context. Several studies have reported positive effects of specific microbial strains, such as *Azotobacter chroococcum*, *Bacillus subtilis*, and *Bacillus megatherium*, on the growth, physiology, and yield of cereal crops (Patruno et al., 2023). For example, improvements in photosynthetic rate, transpiration, stomatal conductance, and grain quality have been observed in maize and wheat following foliar or soil application (Lubyanova et al., 2023). However, these findings are often context-specific and may not be consistently reproducible under different field conditions. The concept of Effective Microorganisms (EM), introduced by Higa and Parr (1994), involves to mixed cultures of beneficial microorganisms intended to modify soil microbial communities. EM formulations typically include lactic acid bacteria and yeasts, phototrophic bacteria, filamentous fungi, and actinomycetes. While EM technology has been proposed as a sustainable alternative to chemical inputs its mechanisms of action and long-term agronomic benefits remain insufficiently understood, and results across studies are inconsistent. Plant growth–promoting microorganisms (PGPMs) are often described as microbial inoculants that can confer benefits to plants through mechanisms such as biological nitrogen fixation, phosphorus solubilization, phytohormone production, pathogen suppression, and induction of stress tolerance (Naik et al., 2020). Although the use of microbial inoculants has increased in both organic and conventional systems, their effectiveness under drought stress conditions is influenced by microbial survival, establishment, and interaction with native soil microbiota. The global production and use of biofertilizers have increased substantially in recent years, with microbial probiotics being increasingly adopted in both organic and conventional farming systems. The application of drought-tolerant PGPM consortia represents an environmentally friendly strategy to alleviate drought stress in cereal crops. Evidence from controlled and field studies suggests that microbial biostimulants can improve certain physiological and agronomic traits in cereals under drought conditions. For instance, barley treated with selected microbial biostimulants exhibit higher relative leaf water content, increased biomass accumulation, improved grain quality and yield, and reduced drought sensitivity (Slimani et al., 2023) (**Table 1**). These beneficial effects are attributed to modifications in root morphology, activation of antioxidant defense systems, and enhanced expression of genes associated with abiotic stress tolerance. However, such benefits are not universal and may differ depending on stress severity and timing. Commercial microbial biostimulants are routinely evaluated under field conditions although variability in experimental design and environmental factors complicates direct comparisons among studies (Jiménez-Gómez et al., 2017; De Souza Vandenberghe et al., 2017). In the Baltic region, newly developed microbial products such as ProbioHumus and NaturGel have been introduced into agricultural practice and are currently available for cereal production. These microbial biostimulants exerted positive effects on wheat and oat growth and productivity. Reported yield increases in wheat and oats, as well as improvements in grain quality parameters, suggest potential agronomic benefits (Gavelienė and Jurkonienė, 2022) (**Table 1**). However, these results are based on specific local conditions and should be interpreted cautiously when extrapolating to other environments. Recent studies on winter wheat indicate that the combined application of probiotic microorganisms and calcium salts activated plant defense responses that partially compensated for the adverse effects of drought stress (Zareyan et al., 2025) (**Table 1**). Similarly, the combined use of EM and vermicompost has been linked to improvements in physiological and biochemical parameters related to drought tolerance, as well as increases in yield and nutrient content (Kanwal et al., 2017; Talaat and Abdel-Salam, 2024a) (**Table 1**). These improvements are likely related to enhanced nutrient mineralization and increased nutrient availability in compost-amended soils. These effects are likely influenced by improved nutrient availability and soil biological activity, although synergistic interactions remain insufficiently characterized. Moreover, current research efforts focus on developing functionally relevant bacterial consortia integrated with humic acid and seaweed extract biostimulants, both of which have been shown to further enhance wheat growth and nutrient accumulation (Pathak et al., 2024) (**Table 1**). While such approaches are promising, their practical effectiveness depends on formulation stability, field performance, and economic feasibility. Overall, microbial biostimulants represent a key strategy for improving drought tolerance, yield stability, and nutrient efficiency in cereal crops. Their application offers a sustainable and cost-effective approach to mitigating productivity losses caused by climate change while reducing reliance on synthetic agrochemicals and optimizing resource use in agroecosystems.

## 10 Conclusions

This review highlights the significant potential of plant biostimulants as sustainable tools for enhancing drought tolerance and maintaining productivity in major cereal crops, including wheat, barley, maize, rice, and oilseed rape. Plant biostimulants represent a promising and sustainable approach for enhancing drought tolerance in cereal crops; however, the strength and consistency of the available evidence differ substantially among biostimulant categories. Strong and consistent evidence across multiple studies supports the effectiveness of several non-microbial biostimulants, particularly humic substances, specific amino acids such as proline and GABA, and selected plant growth regulators. These compounds repeatedly demonstrate positive effects on key physiological processes, including antioxidant defense, osmotic adjustment, and photosynthetic stability, suggesting relatively robust and transferable mechanisms across cereal species and experimental conditions. In contrast, the effectiveness of microbial biostimulants (e.g., PGPR, endophytes, and mycorrhizal fungi), although well-supported mechanistically, appears more variable in practice. Their performance is strongly influenced by environmental conditions, soil characteristics, plant genotype, and microbial strain specificity. As a result, evidence across studies is less consistent, particularly under field conditions. Similarly, certain biostimulant groups, such as seaweed extracts and, in some cases, humic substances, show considerable variability in agronomic outcomes. While many studies report beneficial effects, others indicate neutral or inconsistent responses, highlighting the importance of product composition, application strategy, and environmental context. These findings suggest that their effectiveness should be interpreted cautiously and cannot be universally generalized. Evidence for combined applications of microbial and non-microbial biostimulants indicates potential synergistic effects on plant growth and stress tolerance; however, this area remains insufficiently standardized and is currently supported by a more limited and context-dependent body of research. Overall, while biostimulants clearly contribute to drought stress mitigation through complementary physiological, biochemical, and molecular mechanisms, the reliability of their effects varies. Future research should therefore prioritize distinguishing crop-specific responses, improving product standardization, and validating performance under field conditions. Such efforts are essential to translate promising but sometimes inconsistent findings into reliable and broadly applicable agronomic practices. The ability to reduce reliance on synthetic agrochemicals aligns with the goals of environmentally sustainable agriculture. In addition, effective dissemination of practical guidelines to farmers and agricultural advisors will be essential to fully realize the potential of biostimulants in mitigating drought stress and ensuring stable, high-quality cereal production under changing climatic conditions.
